# Smart gating membranes with *in situ* self-assembled responsive nanogels as functional gates

**DOI:** 10.1038/srep14708

**Published:** 2015-10-05

**Authors:** Feng Luo, Rui Xie, Zhuang Liu, Xiao-Jie Ju, Wei Wang, Shuo Lin, Liang-Yin Chu

**Affiliations:** 1 School of Chemical Engineering, Sichuan University, Chengdu, Sichuan 610065, China; 2 State Key Laboratory of Polymer Materials Engineering, Sichuan University, Chengdu, Sichuan 610065, China

## Abstract

Smart gating membranes, inspired by the gating function of ion channels across cell membranes, are artificial membranes composed of non-responsive porous membrane substrates and responsive gates in the membrane pores that are able to dramatically regulate the trans-membrane transport of substances in response to environmental stimuli. Easy fabrication, high flux, significant response and strong mechanical strength are critical for the versatility of such smart gating membranes. Here we show a novel and simple strategy for one-step fabrication of smart gating membranes with three-dimensionally interconnected networks of functional gates, by self-assembling responsive nanogels on membrane pore surfaces *in situ* during a vapor-induced phase separation process for membrane formation. The smart gating membranes with *in situ* self-assembled responsive nanogels as functional gates show large flux, significant response and excellent mechanical property simultaneously. Because of the easy fabrication method as well as the concurrent enhancement of flux, response and mechanical property, the proposed smart gating membranes will expand the scope of membrane applications, and provide ever better performances in their applications.

Membranes are playing more and more important roles in myriad aspects for sustainable development^1^,^2^,^3^,^4^,^5^. As emerging artificial biomimetic membranes, smart gating membranes with porous substrates and functional gates, whose permeation properties can be dramatically controlled or adjusted by the gates in response to mild chemical and/or physical stimuli in the external environments, are attracting ever-increasing interests from various fields^5^,^6^,^7^. Such smart gating membranes could find myriad applications in numerous fields including water treatment^8^,^9^,^10^, controlled release^6^,^11^, chemical/biological separations^12^,^13^, chemical sensors and valves^14^,^15^, tissue engineering^16^ and so on. Easy fabrication, high flux, significant response and strong mechanical strength are critical for the versatility of such smart gating membranes, because these attributes ensure low cost and easy mass-production as well as satisfactory performances of membranes for practical applications. However, current smart gating membranes are still suffering from complicated and difficult-to-scale-up fabrication process, low flux, poor response or weak mechanical property, which severely limits their applications.

Considerable efforts have been directed at addressing these problems by developing diverse strategies to introduce responsive domains into membrane materials for fabrication of smart gating membranes. All the methods for preparing smart gating membranes can be classified into two main types according to time order of introduction of responsive domains, *i.e.*, the responsive domains can be introduced into membrane materials *after* or *before* membrane formation. It is very popular to fabricate smart gating membranes by introducing responsive domains into membrane materials *after* membrane formation, *i.e.*, responsive domains are introduced into or onto preformed porous membrane substrates by certain modification methods including chemical grafting^5^,^6^,^9^,^12^,^13^,^14^,^17^, and physical coating or pore-filling^10^,^18^,^19^. Although such methods could keep the mechanical property of membrane substrates well, the grafting, coating or pore-filling of responsive domains onto or into membrane pores causes an inherent conflict between the flux and the responsive property. Usually, the more the responsive domains introduced, the more significant the responsive property, but the lower the flux, and *vice versa*. Furthermore, such two-step processes, *i.e.*, preparing porous membrane substrates first and then modifying the membrane substrates with responsive domains, are still somewhat sophisticated and difficult to be scaled up. Therefore, several one-step processes have been developed for fabricating smart gating membranes by introducing responsive domains into membrane materials *before* membrane formation^20^,^21^,^22^,^23^. The responsive domains can be introduced into the membrane-forming materials by either chemical grafting^20^,^21^ or physical blending^22^,^23^, and then the responsive-domain grafted or blended membrane-forming materials are simply processed into smart gating membranes via liquid-induced phase separation (LIPS). The biggest advantage of such one-step methods is that, the smart gating membranes can be mass-produced with the currently existed large-scale industrial equipment for membrane fabrication, *i.e.*, it is very easy for such methods to be scaled up if proper responsive functional polymers or domains are available. However, the LIPS process is very fast during the membrane formation. Generally speaking, it only takes a few seconds for the polymer membranes to finish the solidification during the LIPS process. Thus, the position and distribution of responsive domains in the prepared membranes are difficult to be rationally controlled exactly in such a rapid process, *i.e.*, it is still hard to achieve desired or perfect gates in the membrane pores; as a result, either the responsive property or the flux is limited^20^,^21^,^22^,^23^, which greatly restricts the application and popularization of such methods. Besides, because the introduction of responsive domains causes pores in the membranes during the LIPS process, there exists an inherent conflict between the responsive property and the mechanical property of the prepared membranes. Up to now, easy fabrication of smart gating membranes with simultaneous high flux, significant response and strong mechanical strength still remains a challenge.

Here we report a novel and simple strategy for one-step fabrication of smart gating membranes with simultaneous large flux, significant response and excellent mechanical property by constructing self-assembled responsive nanogels *in situ* on membrane pore surfaces as functional gates *via* a vapor-induced phase separation (VIPS) process. Compared with the LIPS process, the VIPS process is much slower due to the differences from the kinetic terms. Usually, it takes several minutes for the polymer membranes to finish the solidification during the VIPS process. Thus, it is much more flexible at tuning morphology of membranes in VIPS process rather than LIPS process^24^. We demonstrate the membrane fabrication strategy by preparing thermo-responsive gating polyethersulfone (PES) membranes with poly(*N*-isopropylacrylamide) (PNIPAM) nanogels as functional gates, because PNIPAM is a typical thermo-responsive smart material that can undergo a dramatic and reversible volume phase transition around the volume phase transition temperature (VPTT, about 33 °C)^23^,^25^. Typically, the phase transition process of PNIPAM nanogels takes less than a second, showing sufficient potential in rapid response. By simply blending PNIPAM nanogels with PES *before* membrane formation, the PNIPAM nanogels are *in situ* self-assembled on the membrane pore surfaces during the membrane formation process *via* VIPS. The formed membrane pores are three-dimensionally interconnected inside the membranes and the self-assembled PNIPAM nanogels on the membrane pore surfaces serve as thermo-responsive gates, *i.e.*, three-dimensionally interconnected networks of thermo-responsive gates are generated in the membranes. Afforded by such a unique architecture inside the membrane, high flux, significant response and strong mechanical properties of our thermo-responsive gating membranes can be obtained simultaneously without any conflict. Importantly, our one-step method for fabricating smart gating membranes can be easily scaled up, because nowadays responsive nanogels can be prepared simply and controllably^25^.

## Results

### Fabrication of smart gating membranes

**Figure 1:**
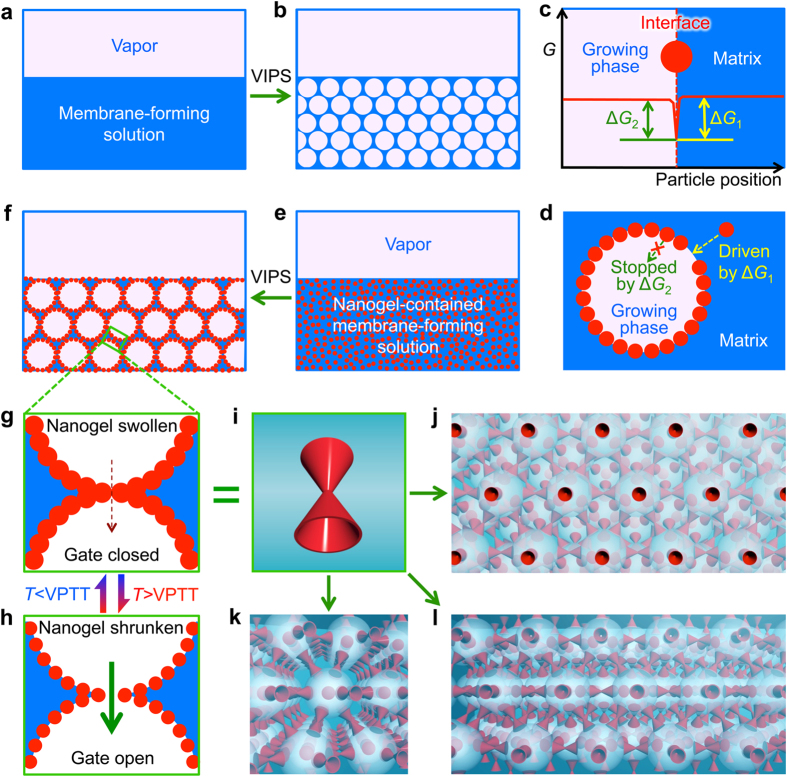
Schematic illustration of design and fabrication of smart gating membranes with self-assembled responsive nanogels as functional gates. (**a**,**b**) Vapor-induced phase separation (VIPS) process for fabricating porous membrane with cellular-like structure (**b**) from homogenous membrane-forming solution (**a**). (**c**,**d**) Principle of self-assembly of nanogels at the growing pore/matrix interface, in which the adsorption of dispersed nanogels in matrix onto the growing pore/matrix interface is driven by a reduction in system interfacial energy (energy well Δ*G*_1_), and the escape of nanogels from the interface to the growing pore phase is stopped by an increase in system interfacial energy (energy barrier Δ*G*_2_). When the nanogels are located at the growing pore/matrix interface, the system interfacial energy is the lowest (**c**); therefore, the nanogels prefer to stay firmly at the growing pore/matrix interface (**d**). (**e**,**f**) Fabrication of porous membranes with self-assembled nanogels on the pore surfaces (**f**) from nanogel-contained membrane-forming solution (**e**) via VIPS approach. (**g**,**h**) Magnified illustration of the thermo-responsive gating function with self-assembled nanogels as gates. When the environmental temperature (*T*) is lower than the volume phase transition temperature (VPTT) of poly(*N*-isopropylacrylamide) (PNIPAM) nanogels (*T*<VPTT), the nanogels are in the swollen state and thus the gate is closed (**g**); on the contrary, when *T*>VPTT, the nanogels are in the shrunken state and thus the gate is open (**h**). (**i**–**l**) 3D graphic illustration of the functional gate (**i**) as well as the top view (**j**) and side views (**k**,**l**) of interconnected networks of functional gates connecting pores inside membrane.

The fabrication procedure for our smart gating membranes is very simple and controllable (Fig. 1). Usually, *via* VIPS processes, porous membranes with symmetric cellular-like structure can be fabricated from homogenous membrane-forming solution (Fig. 1a,b). Nevertheless, the trans-membrane flux of such membranes with symmetric cellular-like structure is very low, because the pores are usually closed and not interconnected with each other^24^. That is, such symmetric cellular-like structures are actually undesirable for common porous membranes. Here, we take advantage of the uniform size and uniform distribution of pores in the symmetric cellular-like structures, and design a simple and controllable strategy to achieve the *in situ* self-assembly of nanogels at the pore/matrix interfaces. Monodisperse PNIPAM nanogels are easily synthesized by precipitation polymerization^23^,^25^, and then blended with PES in the membrane-forming solution using 1-methyl-2-pyrrolidinone (NMP) as solvent. During the VIPS processes with different preparation conditions, the nanogels are self-assembled *in situ* at the growing pore/matrix interfaces. The adsorption of dispersed PNIPAM nanogels in PES matrix onto the growing pore/matrix interface is driven by a reduction in the system interfacial energy (energy well Δ*G*_1_)^26^, and the escape of nanogels from the interface to the growing pore phase is stopped by an increase in the system interfacial energy (energy barrier Δ*G*_2_). When the nanogels are located at the growing pore/matrix interface, the system interfacial energy is the lowest (Fig. 1c); therefore, the nanogels prefer to stay firmly at the growing pore/matrix interface (Fig. 1d). PNIPAM is in the hydrophilic state at 25 °C^23^,^25^; so, the PNIPAM nanogels self-assembled *in situ* at the growing pore/matrix interfaces tend to take in more water into the growing pore spaces. Thus, the size of membrane pores with *in situ* self-assembled nanogels at the interfaces is enlarged. As a result, unlike the cellular-like structure of PES membranes prepared without blending nanogels (Fig. 1a,b), the enlarged pores with *in situ* self-assembled nanogels at the interfaces are interconnected with each other inside the porous membrane that prepared from nanogel-contained membrane-forming solution *via* VIPS approach (Fig. 1e,f). The *in situ* self-assembled PNIPAM nanogels at the interfaces of interconnected pores serve as thermo-responsive gates in the membrane (Fig. 1g,h). When the environmental temperature (*T*) is lower than the VPTT of PNIPAM nanogels (*T*<VPTT), the nanogels are in the swollen state and thus the gate is closed (Fig. 1g); on the contrary, when *T*>VPTT, the nanogels are in the shrunken state and thus the gate is open (Fig. 1h). Because the membrane pores with *in situ* self-assembled nanogels at the interfaces are interconnected with each other, the thermo-responsive smart gates exist like three-dimensionally interconnected gating networks that connect the membrane pores (Fig. 1i–l). Such three-dimensionally interconnected architecture of the pores and the gates can be very beneficial to the concurrent large flux and significant stimuli-response properties of smart gating membranes.

**Figure 2:**
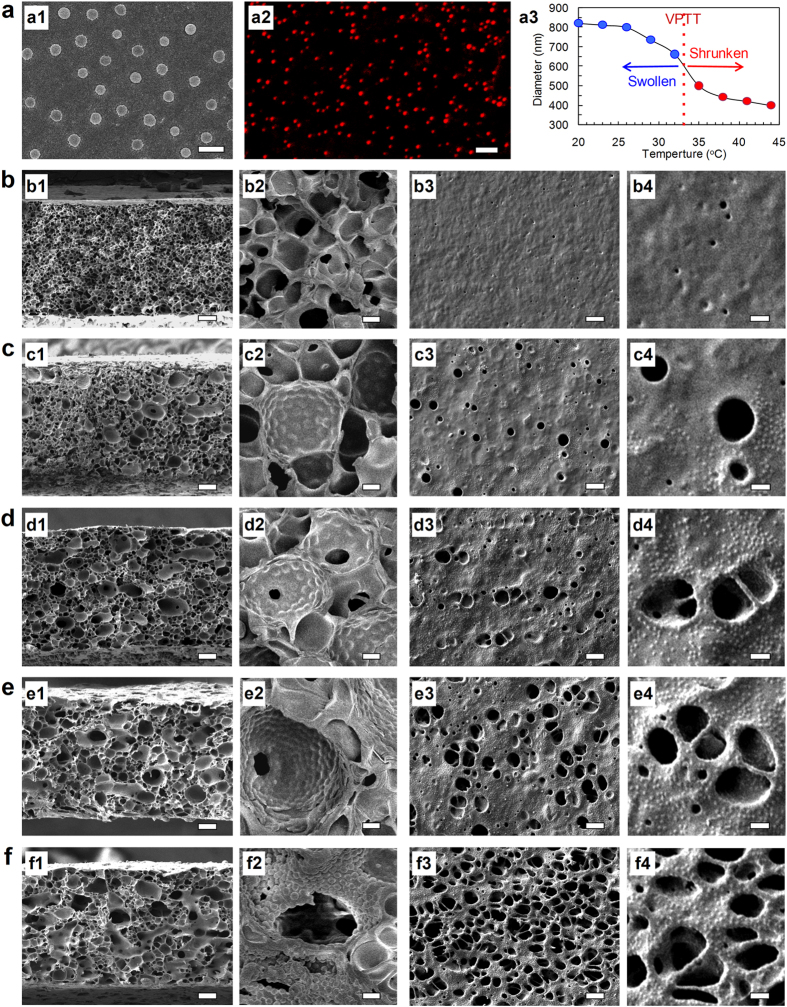
Morphology of nanogels and membranes. (**a**) FESEM image of air-dried PNIPAM nanogels (a1), CLSM image of PNIPAM nanogels dyed with Polyfluor 570 in water at room temperature (a2), and thermo-responsive hydrodynamic diameter of the PNIPAM nanogels in water (a3). Scale bars are 1 μm in (a1) and 3 μm in (a2). (**b**) FESEM images of cross-section (b1 and magnified b2) and surface (b3 and magnified b4) views of the reference membrane prepared via VIPS without nanogels. (**c–f**) FESEM images of cross-section (c1–f1 and magnified c2-f2) and surface (c3–f3 and magnified c4-f4) views of membranes prepared via VIPS with the contents of nanogels varying in the range from 4.25% (**c**), 8.50% (**d**), 12.75% (**e**) and 17.00% (**f**). The exposure time is 20 min, the vapor temperature is 25 °C and the relative humidity is 70%. Scale bars are 10 μm in (b1–f1) and (b3–f3), 1 μm in (b2-f2) and 3 μm in (b4–f4).

The field-emission scanning electron microscope (FESEM) and confocal laser scanning microscope (CLSM) micrographs show that the PNIPAM nanogels fabricated by precipitation polymerization are highly monodisperse both in dried state (Fig. 2a1) and in water (Fig. 2a2), and the dynamic light scattering (DLS) data show that the PNIPAM nanogels exhibit dramatic thermo-responsive volume change in water around 33 °C (Fig. 2a3). The average diameter of air-dried PNIPAM nanogels is 385 nm (Fig. 2a1), while the hydrodynamic diameter of the PNIPAM nanogels in water is 820 nm at 25 °C and 400 nm at 44 °C (Fig. 2a3). After freeze-drying, the PNIPAM nanogels are added into the membrane-forming solution, which is NMP containing 17.5 wt% PES. The blending mass ratios of PNIPAM nanogels to PES, which are defined as the nanogel contents in the membranes, are varied from 4.25% to 17.00% to investigate the effects of nanogel contents on the microstructures and performances of membranes. The nanogel-contained membrane-forming solution is casted into a solution film with thickness of 200 μm on a glass plate inside a humidity chamber maintained at some chosen combination of the vapor temperature and relative humidity. The casted film is kept in the humidity chamber for 2 min or 20 min to achieve the VIPS process thoroughly, and then immersed rapidly in a water bath at 22 °C to form flat membrane. Firstly, after exposing to the vapor at 25 °C and 70% relative humidity for 20 min, FESEM micrographs show that the blending of PNIPAM nanogels significantly affects the microstructures of membrane pores (Fig. 2b–f). As a reference, the PES membrane prepared *via* VIPS without adding any PNIPAM nanogels shows typical symmetric cellular-like structure through the whole thickness of membrane (Fig. 2b1), and both the size and number of pores on the membrane surface are very small (Fig. 2b3). Just as designed and expected, after blending PNIPAM nanogels in the membrane-forming solutions, enlarged pores with nanogels self-assembled *in situ* at the pore/matrix interfaces appear in the membranes (Fig. 2c–f). The magnified FESEM micrographs clearly show that the nanogels assemble orderly at the pore/matrix interfaces (Fig. 2c2–f2), and open pores with very small sizes form at the interconnected points where the adjacent enlarged pores with *in situ* self-assembled nanogels meet (Fig. 2c1–f1), as designed in Fig. 1f–h.

**Figure 3:**
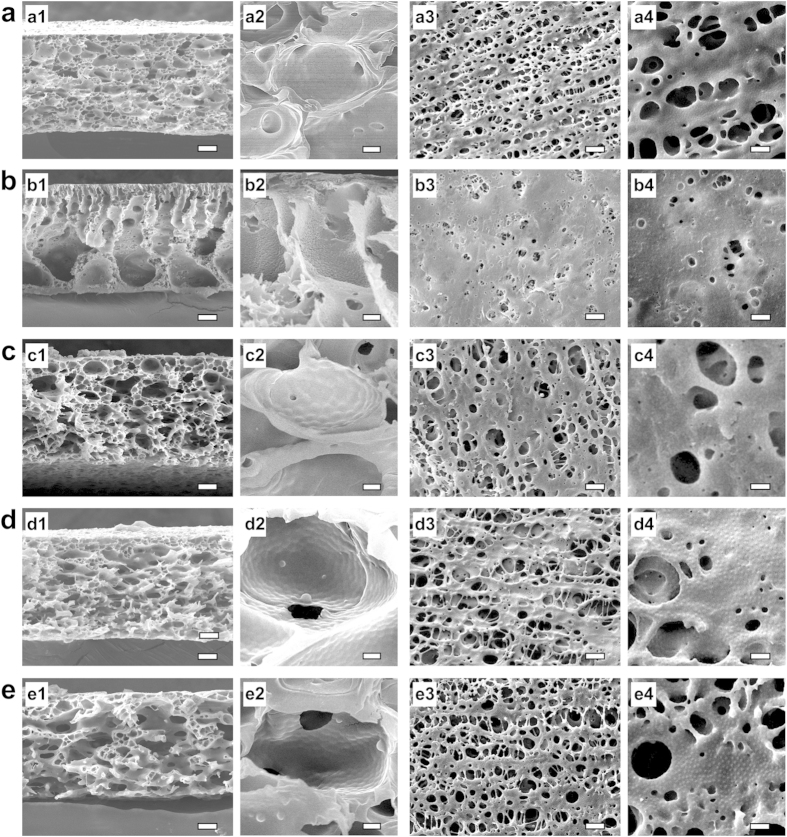
FESEM images of cross-section (a1–e1 and magnified a2–e2) and surface (a3–e3 and magnified a4–e4) views of membranes prepared via VIPS with different conditions. The nanogel content is 17%. (**a**) Exposure time is 2 min, vapor temperature is 25 °C, and relative humidity is 70%. (**b**) Exposure time is 2 min, vapor temperature is 15 °C, and relative humidity is 70%. (**c**) Exposure time is 2 min, vapor temperature is 25 °C, and relative humidity is 90%. (**d**) Exposure time is 20 min, vapor temperature is 25 °C, and relative humidity is 90%. (**e**) Exposure time is 20 min, vapor temperature is 15 °C, and relative humidity is 70%. Scale bars are 10 μm in (a1–e1) and (a3–e3), 1 μm in (a2–e2) and 3 μm in (a4–e4).

In order to optimize the VIPS parameters for membrane preparation, we systematically investigate the effects of the exposure time, the relative humidity and the vapor temperature of VIPS chamber on the microstructure and performance of the membranes. The FESEM images of the membranes are shown in Fig. 3. The exposure process of casting solution in vapor is the primary difference between VIPS and LIPS; therefore, the exposure time should be very important for the membrane formation. Compared with the microstructure of the membrane prepared with exposure time of 20 min and vapor at 25 °C and 70% (RH) (Fig. 2f), that prepared with exposure time of 2 min and vapor at 25 °C and 70% (RH) is significantly different (Fig. 3a). When the exposure time is 20 min, the magnified FESEM micrographs clearly show that a lot of nanogels are observed on the pore/matrix interface and the surface (Fig. 2f2); however, when the exposure time is 2 min, only a few nanogels are observed on the pore/matrix interface and the surface (Fig. 3a2). This phenomenon gives an effective supplement to the formation of the gating structure. The liquid-liquid phase separation occurs in the membrane-forming solution induced by the water vapor, and then the droplets of the polymer-lean phases disperse in the continuous polymer-rich phases. The mild VIPS process gives enough time for the droplets to coarsen. At the same time, the nanogels tend to move to the matrix/growing phase interface due to its hydrophilic property. In this situation, 2 min may be enough for the formation of droplets of the polymer-lean phases, but cannot support the procedure of large number of nanogels moving to the pore/matrix interfaces. Then, we fix the exposure time at 2 min and adjust the vapor temperature to 15 °C and relative humidity to 90% (RH), separately. On the condition of exposure time of 2 min and vapor at 15 °C and 70% (RH), the membrane morphology turns to be finger-like, typical structure from LIPS (Fig. 3b1). The lower temperature slows down the phase separation process, which makes the droplets of the polymer-lean phases hard to coarsen and solidify. Meanwhile, few nanogels appear on the pore/matrix interfaces because the lower temperature slows down the moving velocity of the nanogels (Fig. 3b2). However, on the condition of exposure time of 2 min and vapor at 25 °C and 90% (RH), the membrane pores on the surface are enlarged (Fig. 3c3), which are in accordance with previously reported work^27^. The results show that the exposure time of 2 min is too short for the vapor to influence the membrane formation. Although the vapor temperature and relative humidity varies, the ideal membrane structures cannot be achieved with exposure time of 2 min.

Then, we change the vapor temperature and the relative humidity with fixing the exposure time at 20 min. On the condition of exposure time of 20 min and vapor at 15 °C and 70% (RH), the lower temperature gives the droplets of polymer-lean phases more time to coarsen, so the pore size turns to be larger (Fig. 3e). On the condition of exposure time of 20 min and vapor at 25 °C and 90% (RH), the membrane pores on the surface are also enlarged (Fig. 3d).

To summarize, with increasing the nanogel content from 4.25% to 17.00%, both the number of enlarged pores with *in situ* self-assembled nanogels at the interfaces and that of pores on the membrane surface increase, and the pores become more and more interconnected with each other. The longer exposure time benefits the formation of the designed structure in this study, the lower vapor temperature and higher relative humidity show less significant effects on the formed membrane structure. Considering the principle of convenience and easy-to-scale-up, the mild conditions are better choices. Therefore, the condition of exposure time of 20 min, and the vapor at 25 °C and 70% (RH) are preferred.

The interconnected pores with PNIPAM nanogels self-assembled at the pore/matrix interfaces provide excellent three-dimensionally interconnected gating networks for the membrane to achieve concurrent large flux and significant thermo-responsive characteristics.

### Trans-membrane water flux and thermo-responsive gating characteristics

**Figure 4:**
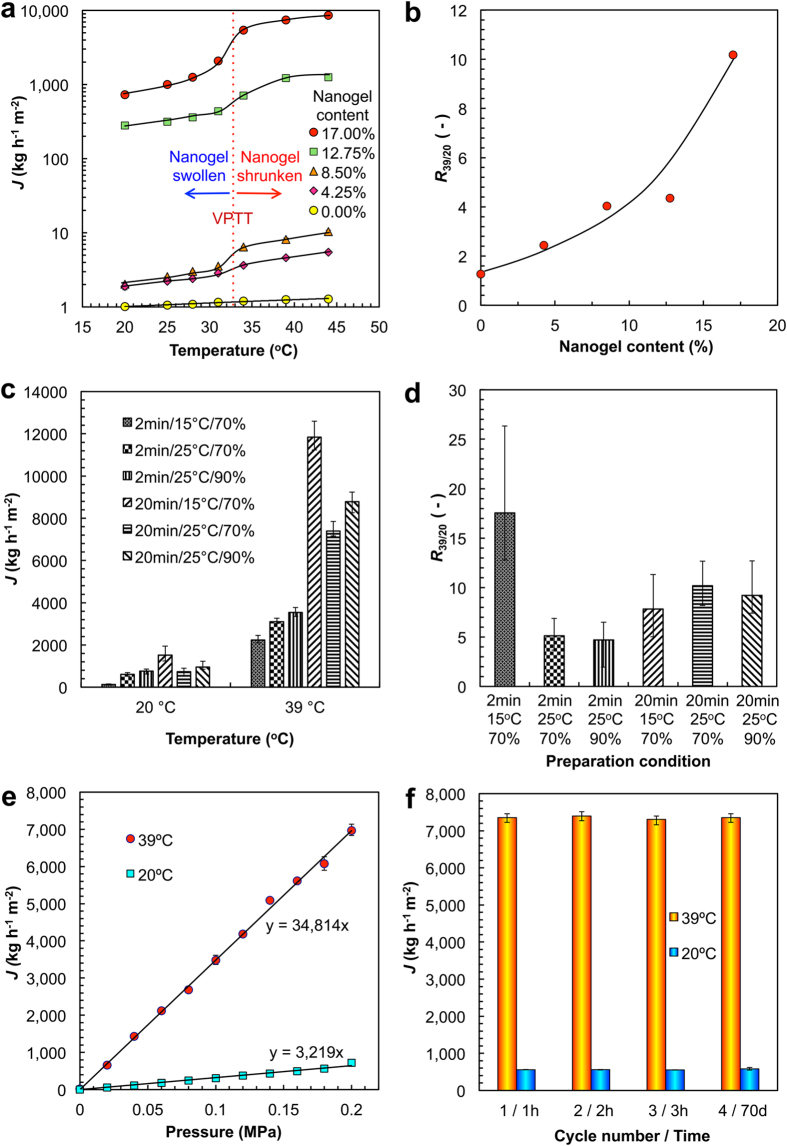
Temperature dependence and reversible thermo-responsive gating characteristics of hydraulic permeability through membranes with nanogel gates. (**a**) Thermo-responsive water fluxes of membranes prepared with different contents of nanogels. The trans-membrane pressure is 0.2 MPa. (**b**) Effect of nanogel content on the thermo-responsive factor of membranes (*R*_39/20_), in which *R*_39/20_ is defined as the ratio of water flux under trans-membrane pressure of 0.2 MPa at 39 °C to that at 20 °C. (**c**) Water fluxes at 20 °C and 39 °C of membranes prepared with different preparation conditions. The trans-membrane pressure is 0.2 MPa. (**d**) Effects of preparation conditions on the thermo-responsive factor of membranes (*R*_39/20_). (**e**) Effect of trans-membrane pressure on the water flux of membrane prepared with nanogel content of 17.00% at 39 °C and 20 °C. (**f**) Reversible thermo-responsive gating characteristics of hydraulic permeability through membranes. The nanogel content is 17.00% and the trans-membrane pressure is 0.2 MPa. Error bars indicate standard deviation, *n* = 5.

Our smart gating membranes with enough *in situ* self-assembled PNIPAM nanogels as thermo-responsive gates show concurrent high flux and significant responsive property in responding to environmental temperature change across the VPTT (Fig. 4). For the reference PES membrane prepared without any nanogels, the trans-membrane water flux is extremely low (Fig. 4a), and the slight increase of the trans-membrane water flux of this membrane with increasing temperature is due to the thermo-induced viscosity decrease of water^6^,^17^. With increasing the nanogel content in the membrane, trans-membrane water flux increases remarkably (Fig. 4a). The results of the trans-membrane water fluxes are in accordance with the microstructures of membranes. As mentioned above, after exposing to the vapor at 25 °C and 70% relative humidity for 20 min, with increasing the nanogel content, both the number of enlarged pores with *in situ* self-assembled nanogels at the interfaces and that of pores on the membrane surface increase, and the pores become more and more interconnected with each other. That is, the more the nanogel content, the larger and the more the trans-membrane pathways for water flow; as a result, the larger the trans-membrane water flux. With the nanogel content of 17.00%, the trans-membrane water flux at 44 °C under operation pressure of 0.2 MPa is as high as 8558 kg h^−1^ m^−2^.

With PNIPAM nanogels self-assembled *in situ* at the pore/matrix interfaces, our membranes show remarkable thermo-responsive characteristics (Fig. 4). A sharp change of water flux appears at temperature near 33 °C, which is the VPTT of PNIPAM nanogels (Fig. 2a3). When the temperature is lower than 33 °C, the nanogels are in the swollen state and the gate is closed (Fig. 1g), as a result the trans-membrane water flux is low; on the contrary, when the temperature is higher than 33 °C, the nanogels are in the shrunken state and the gate is open (Fig. 1h), so the water flux is high (Fig. 4a). To quantitatively characterize the thermo-responsive permeation performance of the membrane, a coefficient called thermo-responsive factor (*R*_39/20_) is defined as the ratio of water flux at 39 °C to that at 20 °C under trans-membrane pressure of 0.2 MPa. The more the nanogel content, the more the PNIPAM nanogels serving as thermo-responsive gates in the membrane, as a result the larger the thermo-responsive factor (Fig. 4b). When the nanogel content is 17.00%, the thermo-responsive factor is as high as 10.2. Trans-membrane water flux and thermo-responsive gating characteristics of membranes prepared by different VIPS parameters are investigated, too. With adjusting the preparation conditions but fixing the nanogel content being 17.00%, the flux and the thermo-responsive characteristics of the membranes vary (Fig. 4c,d). At first, on the condition of exposure time of 2 min and vapor at 15 °C and 70% (RH), the membranes with typical structure from LIPS show obvious different performance from others. Although the thermo-responsive factor is about 17.5, which is higher than others, the flux is only 127 kg h^−1^ m^−2^ at 20 °C and 2228 kg h^−1^ m^−2^ at 39 °C due to the dense surface (Fig. 4c,d). That is, the flux capacity is limited. The other two membranes prepared with exposure time of 2 min, both have a lower thermo-responsive factor around 5 (Fig. 4d), which is corresponding to the imperfect gating structures with few nanogels serving as gates at the pore/matrix interfaces. When the exposure time extends to 20 min, the larger pore size (Fig. 3d,e) brings higher flux (Fig. 4c). From the comparison of both flux and thermo-responsive factor, the condition of exposure time of 20 min and vapor at 25 °C and 70% (RH) is selected as the optimum one (Fig. 4c,d). For the membranes prepared with the condition of exposure time of 20 min and vapor at 25 °C and 70% (RH), the water flux of the membrane increases linearly with increasing the operation pressure at both 39 °C and 20 °C (Fig. 4e), which means that the PNIPAM nanogels assembled at the pore/matrix interfaces are stable enough to resist the experimental pressure and the nanogel gates remain intact in the operation processes. To further confirm the stability of PNIPAM nanogels self-assembled *in situ* at the pore/matrix interfaces, the water that has passed through the membrane is detected by DLS, and the results show that no nanogel is found in the water. Therefore, our smart gating membranes possess excellent reversibility and reproducibility of thermo-responsive performances (Fig. 4f). By alternatively changing the environmental temperature across the VPTT of PNIPAM nanogels repeatedly (20 °C ↔ 39 °C), the trans-membrane water fluxes at both 20 °C and 39 °C keep unchanged even after keeping the membrane in water for 70 days.

Importantly, the trans-membrane water flux and the thermo-responsive property of our smart gating membranes with *in situ* self-assembled nanogels as functional gates can be concurrently enhanced by increasing the nanogel content. By calculating the normalized fluxes and the normalized thermo-responsive coefficients of thermo-responsive membranes with taking into account the effects of operation pressure and temperature-induced viscosity change of water, it is possible to compare the maximum normalized fluxes and thermo-responsive coefficients of membranes prepared with different methods (please see Supplementary Table S1 and Fig. S1 for details). The normalized thermo-responsive coefficient, which is the ratio of membrane resistance at low temperature to that at high temperature, can be used to compare the responsive performances of different membranes at different temperatures directly. For the previous thermo-responsive membranes prepared by introducing thermo-responsive domains into membrane materials *before* membrane formation *via* LIPS, either the maximum normalized fluxes or the maximum normalized thermo-responsive coefficients are limited (Supplementary Table S1 and Fig. S1). For the membranes prepared with grafted thermo-responsive copolymers (“Series 1” in Supplementary Table S1 and Fig. S1), although the maximum normalized fluxes are very large, the maximum normalized thermo-responsive coefficients are not high (typically less than 3.0)^20^. For the membranes prepared by blending membrane-forming materials with thermo-responsive polymers as additives (“Series 2” in Supplementary Table S1 and Fig. S1), both the maximum normalized fluxes (typically lower than 870 L m^−2^ h^−1^ bar^−1^) and the maximum normalized thermo-responsive coefficients (typically less than 1.8) are very limited^21^,^22^. For the membranes prepared by blending membrane-forming materials with thermo-responsive nanogels as additives (“Series 3” in Supplementary Table S1 and Fig. S1), although the maximum normalized thermo-responsive coefficients could be as high as 5.9, the maximum normalized fluxes are very low (typically less than 700 L m^−2^ h^−1^ bar^−1^)^23^. Excitingly, for our membranes prepared *via* VIPS with nanogel content of 17.00%, the maximum normalized flux and the maximum normalized thermo-responsive coefficient are as high as 4300 L m^−2^ h^−1^ bar^−1^ and 6.0 respectively (Supplementary Table S1 and Fig. S1). The results verify that, by constructing the above-mentioned unique architecture inside the membranes *via* VIPS, our smart gating membranes are able to achieve ever better comprehensive performances on the flux and responsive characteristics.

**Figure 5:**
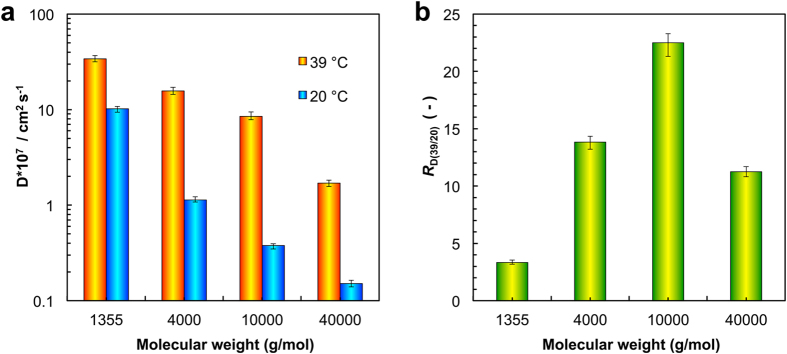
Trans-membrane diffusional permeation performances. (**a**) The thermo-responsive diffusional coefficients of solute molecules with different molecular weights. (**b**) The thermo-responsive diffusion factor (*R*_D(39/20)_) of solute molecules with different molecular weights, in which *R*_D(39/20)_ is defined as the ratio of the diffusion coefficient of the solute at 39 °C to that at 20 °C.

Furthermore, the thermo-responsive gating characteristics of the composite membranes for diffusional permeation of solute molecules with different molecular weights are investigated (Fig. 5, Supplementary Fig. S2, and S3). The results show that the value of the diffusion coefficient of the same solute decreases rapidly with lowering the temperature, which is responding to the changing trend of the flux (Fig. 5a). Then, with increasing the molecular weight of the solute, the diffusion coefficient (*D*) turns down, owing to the increasing of the Stokes-Einstein radius of the solute for diffusion (Fig. 5a).

As mentioned above, for the similar purpose, a coefficient called thermo-responsive diffusion factor (*R*_D(39/20)_) is defined as the ratio of the diffusion coefficient of the solute at 39 °C to that at 20 °C. When the molecular weight of the solute increases from 1355 to 40000, the value of *R*_D(39/20)_ undergoes a process of rising from 3.3 to 22.5 first and then falling to 11.25 later (Fig. 5b). For VB_12_, because the molecular size is small, it is easy for the VB_12_ molecules to permeate through the membrane pores whether the temperature is 39 °C or 20 °C (Supplementary Fig. S3a), and the trans-membrane permeability of VB_12_ is affected by the size change of the diffusion channels to a certain extent. However, for the 4000 and 10000 (MW) FITC-dextrans, at 20 °C, as the molecular size is larger than the “closed” pore size, the molecules are excluded by the membranes; while at 39 °C, the size of the these molecules becomes smaller than the “open” pore size, and then the solute molecules can permeate easily through membranes (Supplementary Fig. S3b). As a result, the value of *R*_D(39/20)_ increases remarkably. In the case of 40000 FITC-dextran with the largest molecule size in this study, even at 39 °C, the permeation of the solute molecule is still affected by the size exclusion of the membrane pores (Supplementary Fig. S3c), because the molecular size is so larger that the 40000 FITC-dextran molecules cannot permeate through the membrane easily. For the solute molecule with molecular weight of 10000 (g/mol), the ratio of the diffusion coefficient of the solute at 39 °C to that at 20 °C is as high as 22.5, which verifies the fabricated membranes are “smart” and highly potential in separations and controlled release.

### Mechanical properties

**Figure 6:**
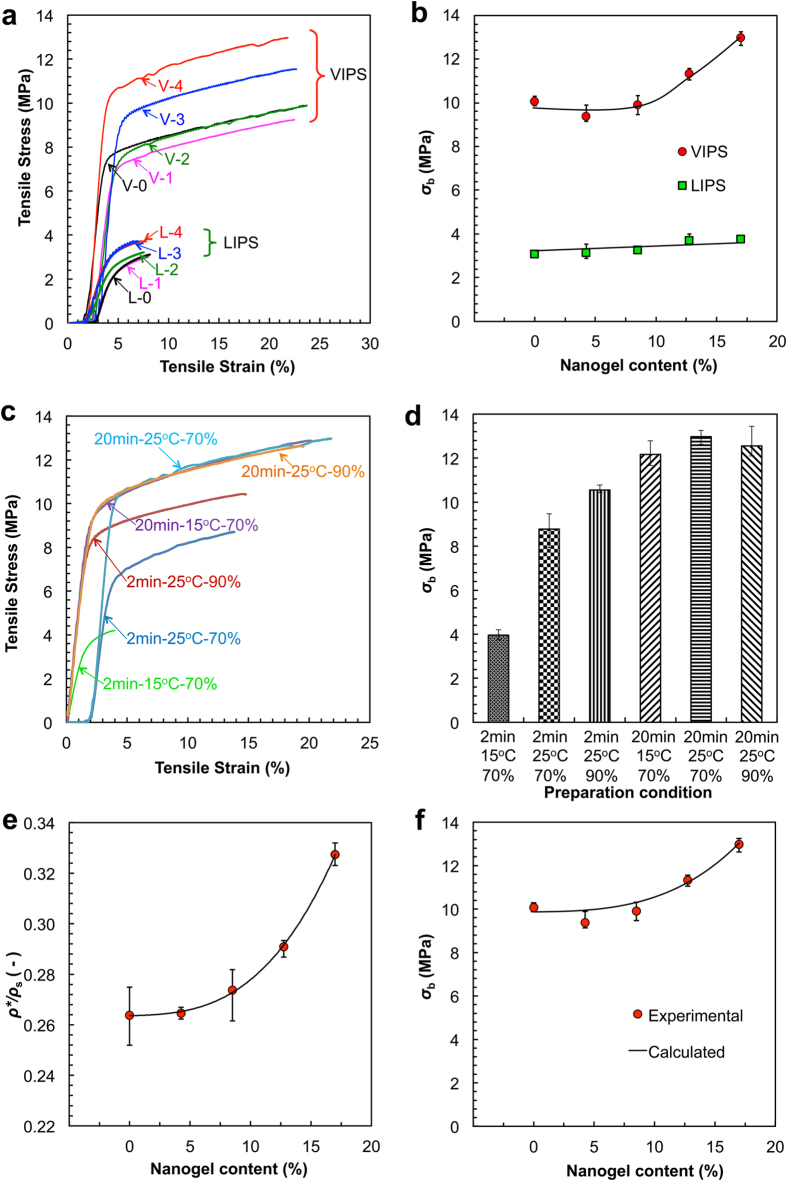
Mechanical properties of membranes. (**a**) Typical tensile stress *versus* tensile strain curves of membranes, in which “V-0” and “L-0” stand for membranes prepared by VIPS and LIPS respectively with nanogel content being 0%, and “V-1” and “L-1” for nanogel content being 4.25%, “V-2” and “L-2” for nanogel content being 8.50%, “V-3” and “L-3” for nanogel content being 12.75% and “V-4” and “L-4” for nanogel content being 17.00%. (**b**) Effect of nanogel content on the tensile strength at break (*σ*_b_) of membranes. (**c**) Typical tensile stress *versus* tensile strain curves of membranes prepared with different conditions. (**d**) Effects of preparation conditions on the tensile strength at break (*σ*_b_) of membranes. (**e**) Effect of nanogel content on the relative density (ρ*/ρ_s_) of membranes, in which ρ* and *ρ*_s_ represent bulk density and true density respectively. (**f**) Comparison of calculated tensile strength at break (*σ*_b_) of membranes from equation (1) with experimental data. Error bars indicate standard deviation, *n* = 5.

Our smart gating membranes with enough *in situ* self-assembled PNIPAM nanogels as thermo-responsive gates exhibit excellent mechanical properties (Fig. 6). On the condition of exposure time of 20 min and vapor at 25 °C and 70% (RH), Our smart gating membranes prepared *via* VIPS have much better mechanical properties than the membranes prepared *via* LIPS (Fig. 6a,b). To compare the mechanical properties of our membranes prepared *via* VIPS with those prepared *via* LIPS, PES membranes with equal contents of nanogels are prepared *via* LIPS as references. Although the thicknesses of casted solution films are all 200 μm, the thicknesses of dried membranes prepared *via* VIPS are 64 ± 4 μm while those prepared *via* LIPS are 98 ± 5 μm. Because the membranes prepared *via* VIPS have symmetric porous structures^24^ while those prepared *via* LIPS have asymmetric porous structures^23^, the membranes prepared *via* VIPS are denser throughout the whole membrane thickness that those prepared *via* LIPS. As a result, the membranes prepared *via* VIPS are mechanically stronger than those prepared *via* LIPS. For the membranes prepared *via* LIPS, no matter how the nanogel content varies, the largest tensile strain at break is less than 8.0% and the largest tensile stress at break (*σ*_b_) is smaller than 3.8 MPa; however, for our membranes prepared *via* VIPS, the tensile strains at break are all about 23.0% and the tensile strengths at break are all higher than 9.4 MPa (Fig. 6a,b). More importantly and surprisingly, with increasing the nanogel content from 4.25% to 17.00%, the tensile strengths at break of our membranes prepared *via* VIPS increase from 9.4 MPa to 13.0 MPa (Fig. 6b). The mechanical properties of membranes prepared with different VIPS parameters are also tested. The membranes prepared with the exposure time of 20 min have higher tensile strengths at break and the tensile strains at break than those prepared with the exposure time of 2 min (Fig. 6c,d). Among the membranes prepared by VIPS, the membranes prepared with 2 min, 15 °C and 70% (RH) have a typical structure like those prepared with LIPS, and have a mechanical property like those prepared by LIPS (Fig. 6a–d). The membranes prepared by higher RH with the limited exposure time of 2 min have a better mechanical property (Fig. 6c,d), which implies that higher RH speeds up the process of pore coarsening. It should be noted that with enough exposure time and fixed nanogel content, the mechanical properties of membranes vary little (Fig. 6c,d).

As mentioned above, our membranes prepared *via* VIPS have symmetric cellular-like structures. For cellular solids, the mechanical properties are mainly affected by the most important structural characteristic parameter that is called the relative density^28^. The relative density of a cellular solid is the density ratio of the cellular material (*i.e.* bulk density ρ*) to the solid of which it is made (*i.e.* true density *ρ*_s_). The smaller the relative density (ρ*/*ρ*_s_) is, the larger the porosity of the porous membrane. With increasing the nanogel content from 4.25% to 17.00%, the ρ*/*ρ*_s_ value of the membrane prepared *via* VIPS increases from 0.26 to 0.33 (Fig. 4e). The results indicate that, by adding more nanogels, although the membrane pores are enlarged and get more interconnected with each other (Fig. 2c–f), the membrane porosity is decreased slightly, which means the pore walls become denser. As a result, the tensile strength at break of the membrane prepared *via* VIPS increases with increasing the nanogel content from 4.25% to 17.00%. Because of the open-cellular structures of the membranes prepared *via* VIPS with addition of enough nanogels, the following equation can be used to calculate the tensile strength of the membrane from the relative density^28^:


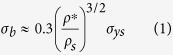


where 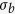 is the tensile strength of the membrane, and 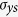 is the yield strength of the pore wall material (PES). The calculated data of the tensile strengths of membranes prepared *via* VIPS with different nanogel contents fit in well with the experimental data (Fig. 6f). Both the experimental and calculated results exhibit an important and exciting phenomenon, which is that the mechanical properties of our smart gating membranes with *in situ* self-assembled nanogels as functional gates are enhanced with increasing the nanogel content. That is, all the flux, responsive and mechanical properties of our smart gating membranes can be simultaneously enhanced without any conflict.

## Discussion

We have demonstrated simple and controllable fabrication of a novel type of smart gating membranes with simultaneous large flux, significant response and excellent mechanical properties, by constructing self-assembled responsive nanogels *in situ* on membrane pore surfaces as functional gates *via* a VIPS process. The generated membrane pores are three-dimensionally interconnected inside the membranes and the self-assembled nanogels on the membrane pore surfaces serve as responsive gates. With the proposed unique architecture, factors conducive to improving all the flux, responsive and mechanical properties are simultaneously introduced into the smart gating membranes. The flux, responsive and mechanical properties of the smart gating membranes can be easily customized by adjusting the nanogel content, and the effects of preparation conditions on the structures and performances of the composite membranes are systematically investigated. By using a proper recipe with enough nanogel content, a smart gating membrane could have all the high flux, significant response and strong mechanical properties. Such a combination of high flux, significant responsive characteristics and strong mechanical properties, along with an easy one-step method of fabrication, makes our smart gating membranes ideal candidates for further investigations and applications. The strategy of self-assembling nanogels *in situ* on the pore surfaces *via* VIPS and the simple fabrication procedure presented here circumvent the difficulties in simultaneously improving flux, responsive and mechanical properties of the smart gating membranes. Due to the excellent concurrent flux, responsive and mechanical properties, the smart gating membranes with *in situ* self-assembled responsive nanogels as functional gates will provide ever better performances in myriad applications including water treatment, controlled release, chemical/biological separations, chemical sensors, chemical valves and tissue engineering, and may open up new fields of application for smart gating membranes. Furthermore, the proposed novel strategy can be used to fabricate various kinds of functional porous materials with pores immobilized or modified by various kinds of responsive or even non-responsive nanoparticles for numerous applications, including smart gating membranes^5^,^6^, anti-fouling membranes^5^,^29^, and functional cellular solids^28^ or foams^30^ and so on, which might be a fertile area of research.

## Methods

### Fabrication of nanogels and membranes

Monodisperse homogenous poly(*N*-isopropylacrylamide) (PNIPAM) nanogels were synthesized by precipitation polymerization^25^. Typically, monomer *N*-isopropylacrylamide (NIPAM), crosslinker *N,N*-methylenebisacrylamide (MBA) and initiator ammonium persulfate (APS) were mixed in a molar ratio of 100:5:2, and dissolved in 200 ml deionized (DI) water with the molar concentration of NIPAM being 0.1 mol L^−1^. To observe the morphology of nanogels in water with confocal laser scanning microscope (CLSM), fluorescence dye methacryloxy thiocarbonyl rhodamine B (Polyfluor 570, Polysciences) was added in the monomer aqueous solution with a concentration of 3.0 mmol L^−1^. The monomer solution was bubbled with nitrogen gas for 30 min to remove the dissolved oxygen, and then was kept in a water bath at 70 °C for precipitation polymerization for 4 h. After reaction, the PNIPAM nanogels were thoroughly purified by repeating centrifugation at 8000 rpm and redispersed in deionized water to remove the residual unreacted components. Finally, the nanogels were freeze-dried at −35 °C for 48 h. The morphology of the PNIPAM nanogels in dried state was observed by field-emission scanning electron microscope (FESEM, JSM-7500F, JEOL). The thermo-responsive hydrodynamic diameters of nanogels in water at temperatures ranging from 20 to 45 °C were measured by dynamic light scattering (DLS, Zetasizer Nano ZS90, Malvern) equipped with a He-Ne light source (λ = 633 nm, 4.0 mW). Before each datum collection, the highly diluted PNIPAM nanogel dispersion in DI water was allowed to equilibrate for 20 min at each predetermined temperature. The morphology of the nanogels dyed with Polyfluor 570 in water at room temperature was observed by CLSM (SP5-II, Leica), with red fluorescent channel excited at 543 nm.

Smart gating membranes with self-assembled responsive nanogels as functional gates were fabricated from nanogel-contained membrane-forming solution via vapor-induced phase separation (VIPS) approach. The membrane-forming solution was 1-methyl-2-pyrrolidinone (NMP) containing 17.5 wt% polyethersulfone (PES, Mw = 40,000, Changchun Jilin Special Plastics). To add the nanogels into the membrane-forming solution, a certain amount of freeze-dried PNIPAM nanogels was dispersed in NMP first, and then PES was added. The nanogel contents in the membranes, which were the blending mass ratios of PNIPAM nanogels to PES, were varied as 0%, 4.25%, 8.50%, 12.75% and 17.00%. The nanogel-contained membrane-forming solution was casted onto a glass plate with a thickness of 200 μm. The casting was performed inside a humidity chamber maintained at 15 °C and 70% relative humidity, 25 °C and 70% relative humidity, and 25 °C and 90% relative humidity, respectively (TH-PE-100, JEIO). The casted film was kept in the humidity chamber for 2 min or 20 min and then immersed in a water bath at 22 °C to form flat membrane. As references, membranes were also prepared with the same recipes via liquid-induced phase separation (LIPS) approach, in which the casted film was immediately immersed into a water bath at 22 °C and left in water for 20 min. The microstructures of membranes were investigated by FESEM (JSM-7500F, JEOL). To observe the cross-sections, membrane samples were put into liquid nitrogen for enough time, fractured mechanically, and stuck to the sample holder. All the samples were sputter-coated with gold for 60 s before observation.

### Thermo-responsive gating property testing

To investigate the thermo-responsive gating characteristics of the prepared membranes, trans-membrane water fluxes at different temperatures were studied first. The water flux experiments of membranes were carried out using a filtration apparatus under a constant trans-membrane pressure of 0.2 MPa. Each membrane had been immersed in DI water over 24 h before testing the water flux. The diameter of the effective membrane area for water permeation was 40 mm. The test temperature range was chosen from 20 °C to 39 °C. In the experiments, a thermostatic unit was used to control the temperatures of the membranes and the feed water. The tests for water flux of each membrane at each temperature were carried out more than five times to obtain an average value for the water flux.

### Mechanical property testing

The mechanical properties of the membranes were tested by a commercial test machine (EZ-LX, Shimadzu). The membrane samples were cut into dumbbell shapes of standardized JIS-K6251-7 sizes (length 35 mm, width 2 mm, and gauge length 12 mm) with a sample-cutting machine (Dumbbell). Both ends of the dumbbell-shaped samples were clamped, and stretched at a constant velocity of 20 mm min^−1^. At least five samples were tested for each membrane.

### Trans-membrane diffusional permeation experiments

Trans-membrane diffusional permeation experiments of composite membranes that prepared with the condition of exposure time of 20 min and vapor at 25 °C and 70% (RH) were carried out. The environmental temperatures were changing between 20 °C and 39 °C. VB_12_ with molecular weight of 1355 (g/mol) and FITC-dextran molecules with number averaged molecular weights of 4000, 10000 and 40000 (g/mol) were chosen as the solute molecules. The feed solution was prepared by dissolving VB_12_ and FITC-dextran molecules in DI water with a concentration of 0.4 mmol L^−1^ (VB_12_) and 50 mg L^−1^ (FITC-dextrans). The diffusional permeation experiments of membranes were carried out by using a standard side-by-side diffusion cell with a thermostatic unit for controlling the environment temperature. Each test membrane was immersed in the permeant solution overnight before beginning the diffusion experiments. The concentration of VB_12_ in the receptor cell at regular intervals was measured by using an UV-vis Spectrometer (UV-1700, Shimadzu) at a wave length of 361 nm. The concentration of FITC-dextran in the receptor cell at regular intervals was measured by using a fluorescent photometer (RF5301PC, Shimadzu), and the excitation and emission wavelength were 480 and 520 nm respectively. Each concentration of the solutes at regular intervals was measured three times, and the arithmetical mean value was calculated. The diffusivity of the solute across the membrane *D*, can also be calculated using a similar equation derived from Fick’s first law of diffusion as follows^31^:


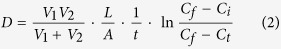


where *C*_i_, *C*_t_ and *C*_f_ are the initial, intermediary (at time *t*), and final concentrations of the solute in the receptor cell; *V*_1_ and *V*_2_ are the volumes of the liquids in the donor cell and in the receptor cell, respectively; *L* represents the thickness of the dry membrane; and *A* is the effective diffusion area of the membrane.

## Additional Information

**How to cite this article**: Luo, F. *et al.* Smart gating membranes with *in situ* self-assembled responsive nanogels as functional gates. *Sci. Rep.* **5**, 14708; doi: 10.1038/srep14708 (2015).

## Supplementary Material

Supporting Information
