# Supplementary material for: Smart gating membranes with in situ self-assembled responsive nanogels as functional gates
Source: Sci Rep. 2015 Oct 5;5:14708. doi: 10.1038/srep14708 (PMC4592958; doi:10.1038/srep14708)
Supplement: Supporting Information [file srep14708-s1.doc]

**Supplementary Information**

**Smart gating membranes with *in situ* self-assembled responsive nanogels as functional gates**

Feng Luo^1^, Rui Xie^1*^, Zhuang Liu^1^, Xiao-Jie Ju^1,2^, Wei Wang^1^, Shuo Lin^1^ & Liang-Yin Chu1,2[[1]](#footnote-2)*

**Table S1 │ Comparison of maximum normalized fluxes and thermo-responsive coefficients of membranes prepared with different methods**

| No. | Membrane formation process | Method to introduce thermo- responsive domains | Membrane formation materials | Formation of thermo-responsive domains | Maximum normalized flux (L m^-2^ h^-1^ bar^-1^) | Maximum normalized thermo-responsive coefficient *N*=*R*_L_/*R*_H_ (-)* | Reference |
| --- | --- | --- | --- | --- | --- | --- | --- |
| 1 | Liquid- induced phase separation  (LIPS) | Prepare membranes with grafted responsive copolymers  (Series 1) | Poly(vinylidene fluoride) (PVDF) | poly(*N*-isopropyl- acrylamide)-graft- poly(vinylidene fluoride) (PVDF- g-PNIPAM) copolymers | 160000 | 2.90 | [S1] |
| 2 |  |  | PVDF | PVDF-g-PNIPAM copolymers | 160000 | 3.00 | [S2] |
| 3 |  |  | PVDF | Blends of poly(acrylic acid)-graft-PVDF copolymers and PNIPAM homopolymers | 200000 | 1.73 | [S3] |
| 4 |  |  | Fluorinated polyimide (FPI) | FPI-g-PNIPAM copolymers | 8250 | 1.46 | [S4] |

| 5 | Liquid- induced phase separation  (LIPS) | Blend membrane- forming materials with thermo- responsive polymers as additives  (Series 2) | PVDF | PVDF-g-PNIPAM copolymers | 870 | 1.04 | [S5] |
| --- | --- | --- | --- | --- | --- | --- | --- |
| 6 |  |  | Polyethersulfone (PES) | poly(NIPAM-co- methacrylic acid-co-methyl methacrylate) terpolymers | 4 | 1.13 | [S6] |
| 7 |  |  | Polyacrylonitrile (PAN) | PAN-g-PNIPAM copolymers | 667 | 1.84 | [S7] |
| 8 |  |  | Solysulfone (PSF) | poly(N-vinylcaprolactam-co-acrylic acid) copolymers | 19 | 1.39 | [S8] |
| 9 | Liquid- induced phase separation  (LIPS) | Blend membrane- forming materials with thermo- responsive nanogels as additives  (Series 3) | PES | PNIPAM nanogels | 700 | 5.94 | [S9] |
| 10 |  |  | PVDF | PNIPAM microgels | 400 | 1.00 | [S10] |

| 11 | Vapor- induced phase separation  (VIPS) | Blend membrane- forming materials with thermo- responsive nanogels as additives  (This work) | PES | PNIPAM nanogels | 4300 | 6.00 | This work |
| --- | --- | --- | --- | --- | --- | --- | --- |

**Note*: The normalized thermo-responsive coefficient (*N*), which is the ratio of membrane resistance at the lowest test temperature (*R*_L_) to that at the highest test temperature (*R*_H_) in the study, is calculated with the following equation:

$N=\frac{R_{L}}{R_{H}}=\frac{\frac{\Delta P_{L}}{\eta_{L}J_{L}}}{\frac{\Delta P_{H}}{\eta_{H}J_{H}}}=\frac{\Delta P_{L}}{\Delta P_{H}}\cdot\frac{\eta_{H}}{\eta_{L}}\cdot\frac{J_{H}}{J_{L}}$ (S1)

where, “∆*P*” is the trans-membrane pressure, “*η*” is the viscosity, and “*J*” is the flux; and the subscripts “L” and “H” represent respectively the lowest test temperature and the highest test temperature. Because the viscosity of water is taken into account, the values of *R* and *N* are both temperature-corrected. With the *N* values of maximum normalized thermo-responsive coefficients, the responsive performances of different membranes at different temperatures can be compared directly.

**Supplementary References**

1. Ying, L., Kang, E. T. & Neoh, K. G. Synthesis and characterization of poly(*N*-isopropylacrylamide)-*graft*-poly(vinylidene fluoride) copolymers and temperature-sensitive membranes. *Langmuir* **18**, 6416-6423 (2002).
2. Ying, L., Kang, E. T., Neoh, K. G., Kato, K. & Iwata, H. Novel poly(*N*-isopropylacrylamide)-graft poly(vinylidene fluoride) copolymers for temperature-sensitive microfiltration membranes. *Macromol. Mater. Eng.* **288**, 11-16 (2003).
3. Ying, L., Kang, E.T. & Neoh, K.G. Characterization of membranes prepared from blends of poly(acrylic acid)-graft-poly(vinylidene fluoride) with poly(N-isopropylacrylamide) and their temperature and pH-sensitive microfiltration. *J. Membr. Sci.* **224**, 93-106 (2003).
4. Wang, W. C., Ong, G. T., Lim, S. L., Vora, R. H., Kang, E. T. & Neoh, K. G. Synthesis and characterization of fluorinated polyimide with grafted poly(*N*-isopropylacrylamide) side chains and the temperature-sensitive microfiltration membranes. *Ind. Eng. Chem. Res.* **42**, 3740-3749 (2003).
5. Yu, J. Z., Zhu, L. P., Zhu, B. K. & Xu, Y. Y. Poly(*N*-isopropylacrylamide) grafted poly(vinylidene fluoride) copolymers for temperature-sensitive membranes. *J. Membr. Sci.* **366**, 176-183 (2011).
6. Li, H., Liao, J., Xiang, T., Wang, R., Wang, D., Sun, S. & Zhao C. Preparation and characterization of pH- and thermo‐sensitive polyethersulfone hollow fiber membranes modified with P(NIPAAm-MAA-MMA) terpolymer. *Desalination* **309**, 1-10 (2013).
7. Fei, Z. D., Wan, L. S., Wang, W. M., Zhong, M. Q. & Xu Z. K. Thermo-responsive polyacrylonitrile membranes prepared with poly(acrylonitrile-g-isopropylacrylamide) as an additive. *J. Membr. Sci.* **432**, 42-49 (2013).
8. Sinha, M. K. & Purkait M. K. Preparation and characterization of stimuli-responsive hydrophilic polysulfone membrane modified with poly(*N*-vinylcaprolactam-co-acrylic acid). *Desalination* **348**, 16-25 (2014).
9. Wang, G., Xie, R., Ju, X. J. & Chu, L. Y. Thermo-responsive polyethersulfone composite membranes blended with poly(*N*-isopropylacrylamide) nanogels. *Chem. Eng. Technol.* **35**, 2015-2022 (2012).
10. Chen, X., Bi, S., Shi, C., He, Y., Zhao, L. & Chen L. Temperature-sensitive membranes prepared from blends of poly(vinylidene fluoride) and poly(*N*-isopropylacrylamides) microgels. *Colloid Polym. Sci.* **291**, 2419-2428 (2013).


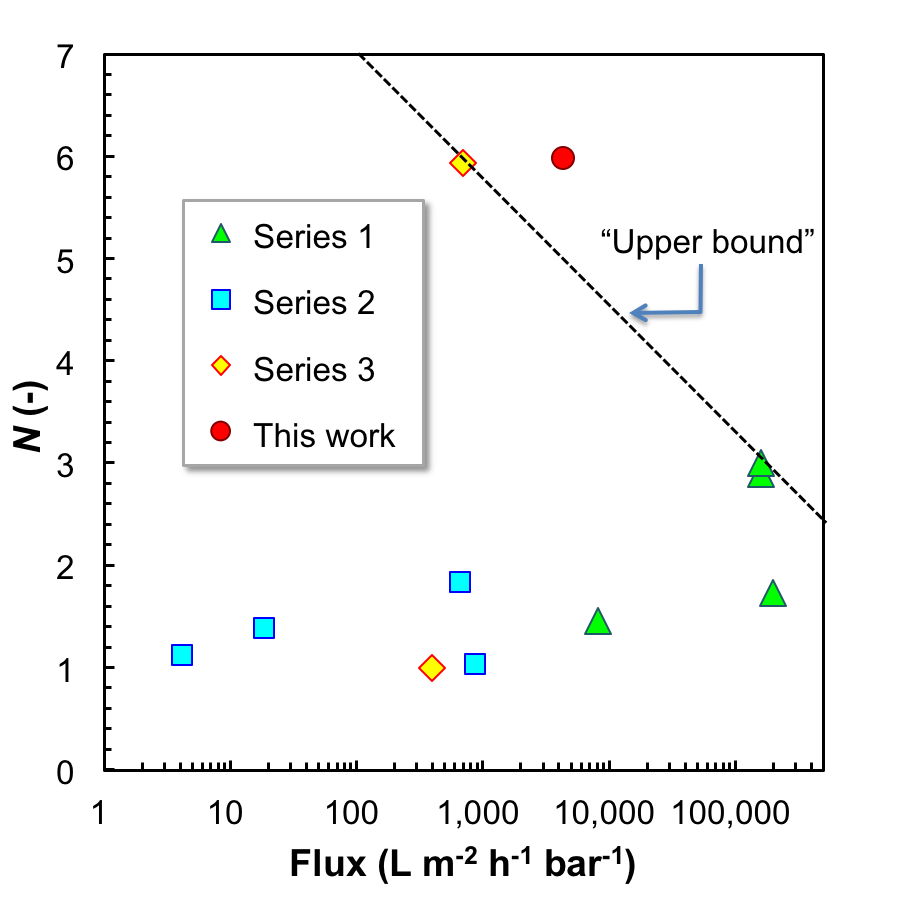


**Figure S1 │ Comparison of maximum normalized fluxes and thermo-responsive coefficients of membranes prepared with different methods** (Data source: please see **Table S1** for details)**.**


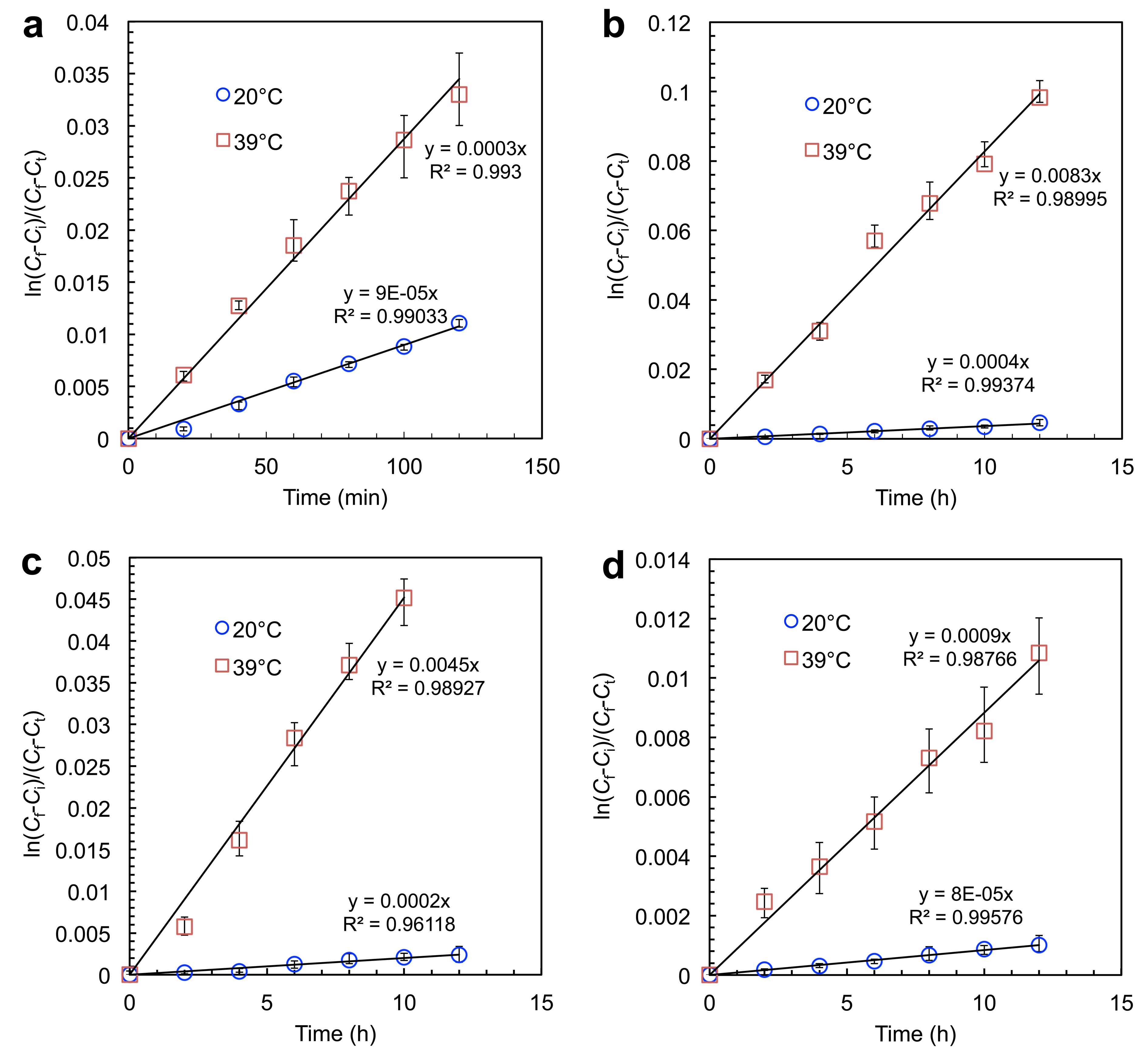


**Figure S2 │ Plot of ln[(*C*_f_-*C*_i_)/(*C*_f_-*C*_t_)] versus *t* of different solutes across the membrane at different temperatures. a**, VB_12_ with molecular weight of 1355; **b**, FITC-dextran with molecular weight of 4000; **c**, FITC-dextran with molecular weight of 10000; **d**, FITC-dextran with molecular weight of 40000.


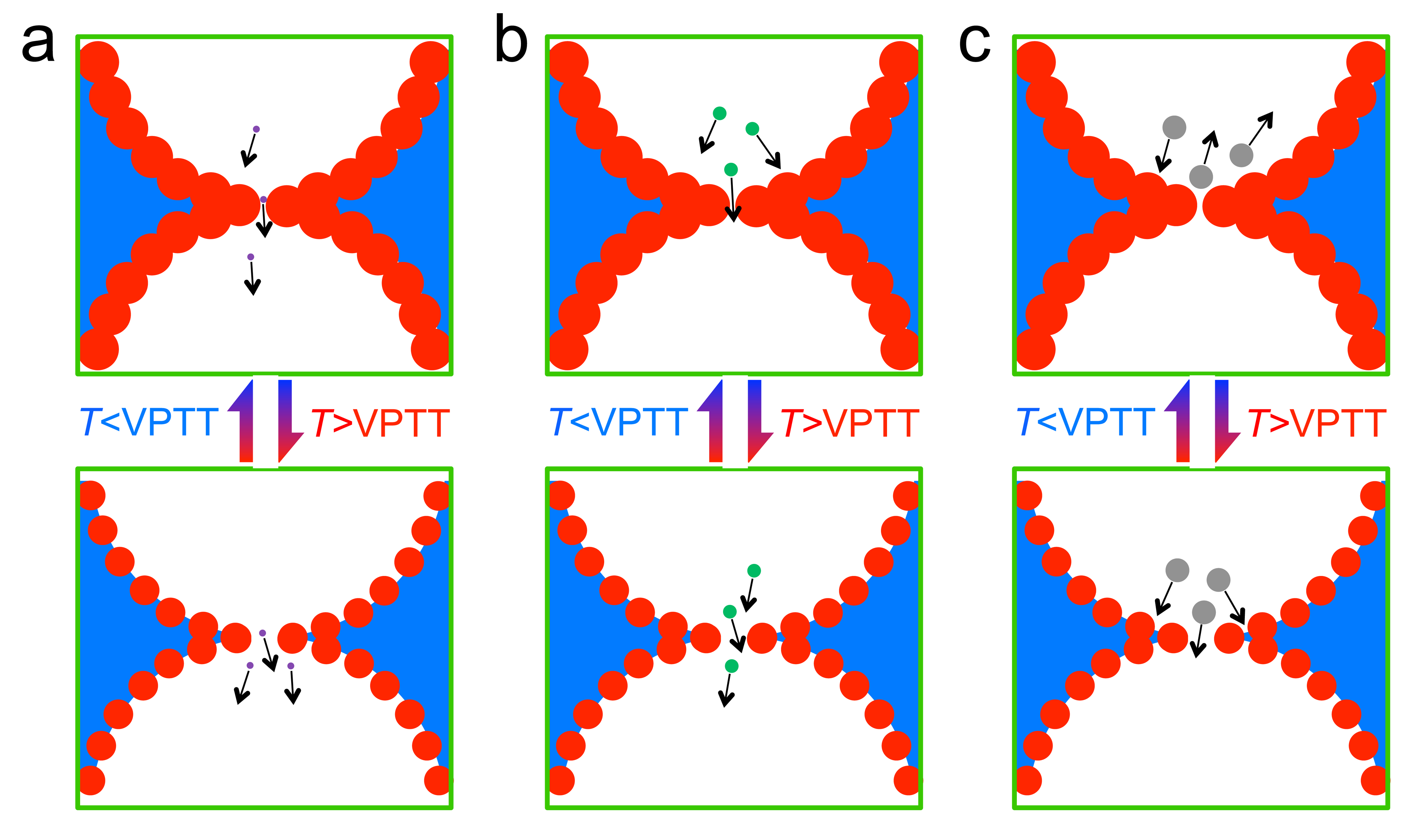


**Figure S3 │ Schematic illustration of thermo-responsive diffusional permeation characteristics of solutes with small (a), medium (b) and large (c) molecular weights across the gating membrane.**

1. *^1^School of Chemical Engineering, Sichuan University, Chengdu, Sichuan 610065, China. ^2^State Key Laboratory of Polymer Materials Engineering, Sichuan University, Chengdu, Sichuan 610065, China. Correspondence and requests for materials should be addressed to R.X. (email: [xierui@scu.edu.cn](mailto:xierui@scu.edu.cn)) or to L.-Y.C. (email: [chuly@scu.edu.cn](mailto:chuly@scu.edu.cn)). [↑](#footnote-ref-2)
